# Alterations in exosomal miRNA profile upon epithelial-mesenchymal transition in human lung cancer cell lines

**DOI:** 10.1186/s12864-018-5143-6

**Published:** 2018-11-06

**Authors:** Yue-Ting Tang, Yi-Yao Huang, Jing-Huan Li, Si-Hua Qin, Yong Xu, Tai-Xue An, Chun-Chen Liu, Qian Wang, Lei Zheng

**Affiliations:** 1 Department of Laboratory Medicine, Nanfang Hospital, Southern Medical University, No.1838 North Guangzhou Avenue, Guangzhou, 510515 Guangdong China; 2 Department of Clinical Laboratory, Zhujiang Hospital, Southern Medical University, Guangzhou, Guangdong China; 3 Department of Clinical Laboratory, Zhongnan Hospital, Wuhan University, Wuhan, Hubei China; 4 Department of Physiology, Anatomy and Genetics, University of Oxford, Oxford, Oxfordshire UK

**Keywords:** Exosomes, Epithelial mesenchymal transition, miRNA, Lung cancer, Metastasis, High-throughput sequencing

## Abstract

**Background:**

Epithelial–mesenchymal transition (EMT) is regarded as a critical event during tumor metastasis. Recent studies have revealed changes and the contributions of proteins in/on exosomes during EMT. Besides proteins, microRNA (miRNA) is another important functional component of exosomes. We hypothesized that the miRNA profile of exosomes may change following EMT and these exosomal miRNAs may in return promote EMT, migration and invasion of cancer cells.

**Results:**

The small RNA profile of exosomes was altered following EMT. Kyoto Encyclopedia of Genes and Genomes (KEGG) pathway analysis revealed that the specific miRNAs of M-exosomes have the potential to drive signal transduction networks in EMT and cancer progression. Co-culture experiments confirmed that M-exosomes can enter epithelial cells and promote migration, invasion and expression of mesenchymal markers in the recipient cells.

**Conclusion:**

Our results reveal changes in the function and miRNA profile of exosomes upon EMT. M-exosomes can promote transfer of the malignant (mesenchymal) phenotype to epithelial recipient cells. Further, the miRNAs specifically expressed in M-exosomes are associated with EMT and metastasis, and may serve as new biomarkers for EMT-like processes in lung cancer.

**Electronic supplementary material:**

The online version of this article (10.1186/s12864-018-5143-6) contains supplementary material, which is available to authorized users.

## Background

Lung cancer is one of the most common and lethal cancers worldwide [1], and metastasis is the leading cause of lung cancer-related deaths [2], which highlights the urgent need to better understand the critical steps and mechanisms of metastasis. Exosomes are small membrane vesicles (30–150 nm) secreted by a variety of cells. They are spherical particles enclosed by a phospholipid bilayer, containing DNA, RNA, and protein, and are released into the extracellular environment [3]. It has been shown that exosomes are local and systemic cell-to-cell mediators of information, through the horizontal transfer of signaling macromolecules, in various pathophysiological processes [4, 5]. Tumor cells may secrete large amounts of exosomes, which are currently considered one of the main contributors to tumor progression and metastasis by promoting the proliferation and inhibiting apoptosis of tumor cells [6], activating angiogenic pathways [7], and enhancing invasiveness and migration of tumor cells [8, 9].

Epithelial–mesenchymal transition (EMT), a process in which epithelial cells (E-cells) lose their polarity and are converted into mesenchymal cells (M-cells), is regarded as a critical event during tumor metastasis [10, 11]. Several studies have revealed the contribution of exosomes during the EMT process in various cancers [12, 13]. On one hand, tumor-derived exosomes transfer specific cargoes to recipient cells, leading to the process of EMT and tumor development [14–17]. On the other hand, the profiles of exosomal proteins and RNAs may also be altered following EMT [18–20]. Thus, the contents of exosomes may be used as new biomarkers to monitor the process of EMT and tumor development.

Previous studies on exosomes have focused on the changes in the exosomal proteome during EMT [18, 20–22]. For example, the mRNA and protein levels of vimentin in exosomes were found to be increased after EMT [17]. However, microRNAs (miRNAs), another important functional component of exosomes, have rarely been reported in studies of EMT in lung cancer. Only one study has reported that the level of miR-23a was increased in exosomes secreted by mesenchymal lung cancer cells [23]. Given that multiple specific miRNAs may be secreted in tumor-derived exosomes and that they may also be altered during tumor progression [24, 25], it is worth investigating the changes in the overall exosomal miRNA profile during the EMT process. Hence, for the first time, we comprehensively analyzed changes in the small RNA (sRNA) profiles of exosomes following EMT of lung cancer cells using high-throughput sequencing. Considering that miRNAs transferred by exosomes can alter the behavior of recipient tumor cells to promote oncogenesis [25, 26], we also compared the function of exosomes derived from E-cells with that of exosomes from M-cells carrying different sRNA cargoes, to gather evidence that reprogramming of the miRNA profile of exosomes may accelerate cancer malignancy.

## Methods

### Cell culture and establishment of EMT cell model

The human lung cell line A549 and H1299 provided by Cell bank, Type culture collection, Chinese Academy of Science (CBTCCCAS) and human airway epithelial cell line 16HBE (kindly provided by the Chronic Airways Diseases Laboratory, Nanfang Hospital, Southern Medical University, Guangzhou) were cultured in Roswell Park Memorial Institute (RPMI-1640) medium, 10% fetal bovine serum (FBS) (System Biosciences, Mountain View, CA, USA). Transforming growth factor-β1 (TGF-β1) (R&D Systems, Minneapolis, Minnesota, USA) was dissolved with 4 mM HCL and diluted in phosphate buffer solution (PBS) before use. A549 and H1299 cells were incubated with fresh medium in the presence or absence of TGF-β1 after 12 h starvation. The most appropriate stimulating condition (concentration of TGF-β1 (0, 2, 5 ng/ml) and duration of treatment (0, 24, 48 h)) was confirmed by the result of functional studies and evaluation of EMT-related markers. The A549 and H1299 cells untreated with TGF-β (PBS, 48 h) were defined as E-phenotype cancer cells, and those treated with TGF-β (5 ng/ml TGF-β, 48 h) were defined as M-phenotype cancer cells.

### Cell culture medium (CCM) preparation and exosomes isolation

When the same initial number (2 × 10^5^/ml CCM) of E/M phenotype A549, H1299 and 16HBE cells were grown to 70–80% confluence, the FBS-containing media was removed and cells were cultured in serum-free 1640 medium with 2% Exo-FBS™ exosome-depleted FBS (System Biosciences) for 48 h. Then the CCM samples (100 ml per sample), were collected and centrifuged at 2000×*g* for 10 min and then filtered through 0.22-μm membranes to remove dead cells, cell debris and large particles (shedding vesicles and apoptotic bodies). ExoQuick-TC (System Biosciences) was used for exosomes isolation, according to the manufacturer’s instructions. All centrifugations were performed at 4 °C. The experiment was repeated three times using three completely independent sets of samples (three independent CCM samples prepared at different times). CON-exo, E1-exo, M1-exo, E2-exo, M2-exo represent exosomes derived from 16HBE, E-phenotype A549 cells, M-phenotype A549 cells, E-phenotype H1299 cells, M-phenotype H1299 cells, respectively.

### Nanoparticle tracking analysis (NTA)

Exosome suspensions with concentrations between 1 × 10^7^/ml and 1 × 10^9^/ml were verified using a Nanosight NS300 (Malvern, Great Malvern, UK) equipped with a 405 nm laser to determine the size and quantity of particles isolated. A video of 60 s duration was taken with a frame rate of 30 frames/s, and particle movement was analyzed by NTA software (version 2.3, NanoSight).

### Transmission electron microscopy (TEM)

Aliquots of 20–40 μl of a solution of exosomes were placed on a copper mesh and post-negatively stained with 2% phosphotungstic acid solution for 10 min. Subsequently, the samples were dried for 2 min under incandescent light. The copper mesh was observed and photographed under a HITACHI H-7650 transmission electron microscope (Hitachi High-Technologies, Tokyo, Japan).

### Western blot analysis

Exosomes or cell protein supernatants were denatured in 5 × SDS buffer and subjected to western blot analysis (10% SDS–polyacrylamide gel electrophoresis; 50 μg protein per lane) using rabbit polyclonal antibodies against E-cadherin, N-cadherin, vimentin (Cell Signaling, Danvers, MA, USA), CD9 and CD63 (Santa Cruz, CA, USA), TSG101 (Sigma, Dorset, UK) and calnexin (Bioworld Technology, MN, USA). The proteins were visualized on the Bio-Rad ChemiDoc XRS Imager system (Bio-Rad Laboratories, California, USA).

### Wound healing assays

Cells were wounded using a 200-μl sterile pipette tip. Subsequently, the cells were washed twice with PBS and treated with TGF-β1. The width of each wound was measured and recorded 0, 24 and 48 h after the scratches were made.

### Migration and Matrigel invasion assays

The Matrigel was uncoated (migration assay) or coated (invasion assay) on the upper surface of a transwell chamber (BD Biosciences, Franklin Lakes, New Jersey, USA), and 6 × 10^5^ cells in serum-free medium containing TGF-β1 or exosomes were placed into the upper chamber. The chambers were then incubated in the lower chamber containing culture medium with 10% FBS for 24 h. The number of cells adhering to the lower membrane was observed using an Olympus BX50 microscope (Tokyo, Japan) and digitized using ImageJ software (NIH Image).

### Isolation of exoRNA and cell RNA, and RNA analysis

TRIzol-LS Reagent (Ambion, Life Technology, Carlsbad, CA, USA) was used to isolate high-quality total RNA from exosomes solution. The RNA concentration was assessed using a Quibit 2.0 Fluorometer (Invitrogen, Life Technology, Carlsbad, CA, USA). The RNA yield and size distribution were analyzed using an Agilent 2100 Bioanalyzer with RNA 6000 Pico Kit (Agilent Technologies, Foster City, CA, USA). Cell RNA was extracted using the TRIzol Reagent (TaKaRa, Dalian, China). The mRNA level of EMT markers in the cells was analyzed using a PrimeScriptTM quantitative real-time reagent Kit (TaKaRa).

### Small RNA sequencing

To investigate differences in the miRNA profile among exosomes from E-phenotype and M-phenotype A549 cells, and 16HBE cells, sRNA high-throughput sequencing technology was used. The flowchart of sequencing group preparation is shown in Additional file 2: Figure S2A. Samples of 100 ng of 18 exoRNA or cell RNA were used for RNA library preparation, following the instructions of the TruSeq® sRNA Sample Prep Kit (Illumina, San Diego, CA, USA). Subsequently, the PCR-amplified cDNA construct from 140 to 160 bp was purified. The purified cDNA was directly sequenced using an Illumina HiSeq 2500 platform, and the 3′ adaptor sequences within the read sequences were cleaned up. Clean data were obtained by filtering out low-quality reads. All clean tags were filtered and aligned with the NCBI GeneBank and miRBase databases.

### Exosomes labeling and tracking in A549, H1299 and 16HBE cells

Purified exosomes were labeled with the PKH67 Green Fluorescent Labeling Kit (Sigma) according to the manufacturer’s recommendations. PKH67-stained exosomes were incubated with recipient cells at 37 °C for 24 h. The cells were stained with DAPI (GeneCopoeia, Rockville, MD, USA) and visualized using an Olympus IX71 microscope.

### Exosomes co-culture assay

Different concentrations of exosomes (0, 50, 100 μg/ml) from E-phenotype or M-phenotype cells were co-cultured with 16HBE, A549 and H1299 cells at 37 °C for 48 h. After treatment, functional assays were conducted on recipient cells and the mRNA and protein from recipient cells were extracted and further investigated.

### Statistical analysis

Values are expressed as mean ± standard deviation. All experiments were carried out in triplicate and repeated at least twice. Student’s t-test and an Analysis of Variance (ANOVA) were used to determine the differences between groups. SPSS 15.0 was used for statistical analyses (SPSS Incorporated, Chicago, IL, USA), and *P* < 0.05 was considered to be statistically significant.

## Results

### A549 cells undergo EMT after exposure to TGF-β1

**Fig. 1:**
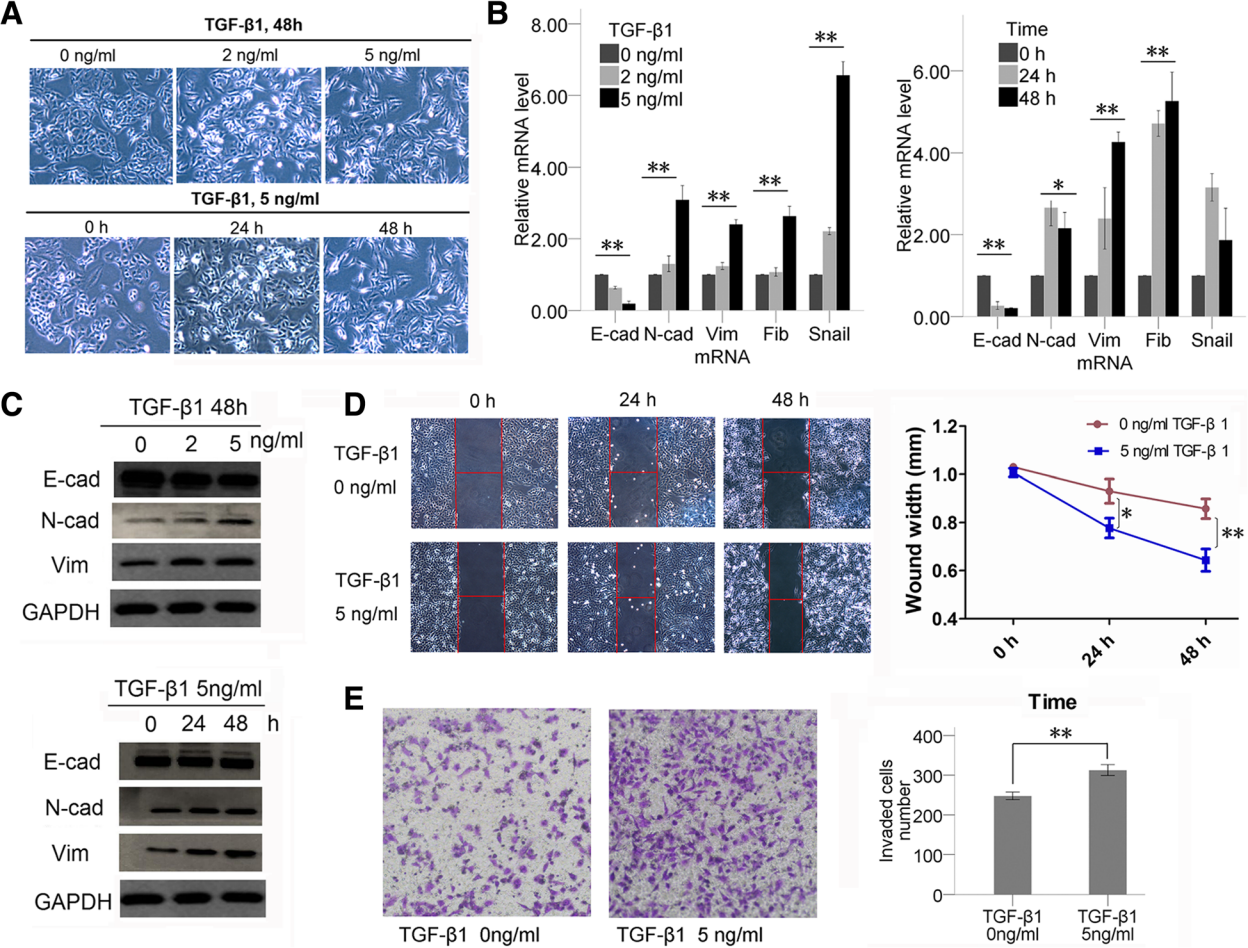
TGF-β1 was used to establish EMT cell models. **a** Morphology of A549 cells changed from E- to M- phenotype after TGF-β1 treatment. The mRNA (**b**) and protein (**c**) levels of EMT-related markers of A549 cells changed after being induced by TGF-β1. TGF-β1 significantly reduced E-phenotype marker (E-cadherin (E-cad)) levels, but increased the M-phenotype marker (N-cadherin (N-cad), vimentin (Vim), fibronectin (Fib) and snail) levels in a TGF-β-concentration-dependent manner but not a strict time-dependent manner. **d** The wound healing assays proved that TGF-β1 treatment can significantly increase cell migration abilities. The wound widths were significantly shorter at 24 h after TGF-β1 treatment than in the no-treatment group (*P* < 0.05), and this difference was more significant after 48 h treatment (*P* < 0.01). **e** Invasion assays were used to determine cell invasion. Invaded cell numbers were significantly higher in TGF-β1 treatment than no-treatment groups, indicating TGF-β1 can improve cell invasion ability

When the A549 cells were treated with TGF-β1 at different concentrations (0, 2, 5 ng/ml) for different durations (0, 24, 48 h), the morphology, function, and expression of EMT markers of cells was changed. Morphologically, A549 cells turned from being round into a spindle-like mesenchymal phenotype and lost intercellular junctions after TGF-β1 treatment in a time- and concentration-dependent manner, which was most prominent after stimulation by 5 ng/ml TGF-β1 for 48 h (Fig. 1a). The process of EMT after TGF-β1 stimulation was also accompanied by a decrease in known epithelial marker (E-cadherin) and increased mesenchymal marker (N-cadherin, vimentin, fibronectin and snail) levels (Fig. 1b, c). The mRNA level (Fig. 1b) and protein level (Fig. 1c) of EMT-related markers changed in a concentration-dependent manner but not a strict time-dependent manner and the most significant change was found when stimulated with 5 ng/ml TGF-β1 for 48 h. The wound healing and invasion assays showed that 5 ng/ml TGF-β1 induced EMT was associated with increased cell migration (Fig. 1d) and invasion (Fig. 1e). In summary, A549 cells stimulated by 5 ng/ml TGF-β1 for 48 h showed the most significant EMT characteristics, so this stimulation condition was chosen to establish the EMT cell model. In the next study, A549 cells untreated with TGF-β1 were defined as E-phenotype cells (PBS, 48 h), and those treated with TGF-β (5 ng/ml, 48 h) were defined as M-phenotype cells.

### Biochemical characterization of exosomes from CCM

To demonstrate the presence of exosomes, the vesicles isolated from A549 CCM were determined by TEM (Additional file 1: Figure S1A), western blotting (Additional file 1: Figure S1B) and NTA (Additional file 1: Figure S1C). A lipid bilayer structure around 100 nm observed by TEM was consistent with descriptions of exosomes (Additional file 1: Figure S1A). The three exosomal marker proteins (CD9, CD63 and TSG101) were present in all vesicle samples (Additional file 1: Figure S1B). NTA demonstrated that all particles were smaller than 300 nm and that most of them were about 50–200 nm in size (Additional file 1: Figure S1C). Additionally, there were no significant differences in the number and average size of exosomes derived from E-phenotype and M-phenotype A549 cells (Additional file 1: Figure S1D).

### sRNA composition in cells differs from that in exosomes

**Fig. 2:**
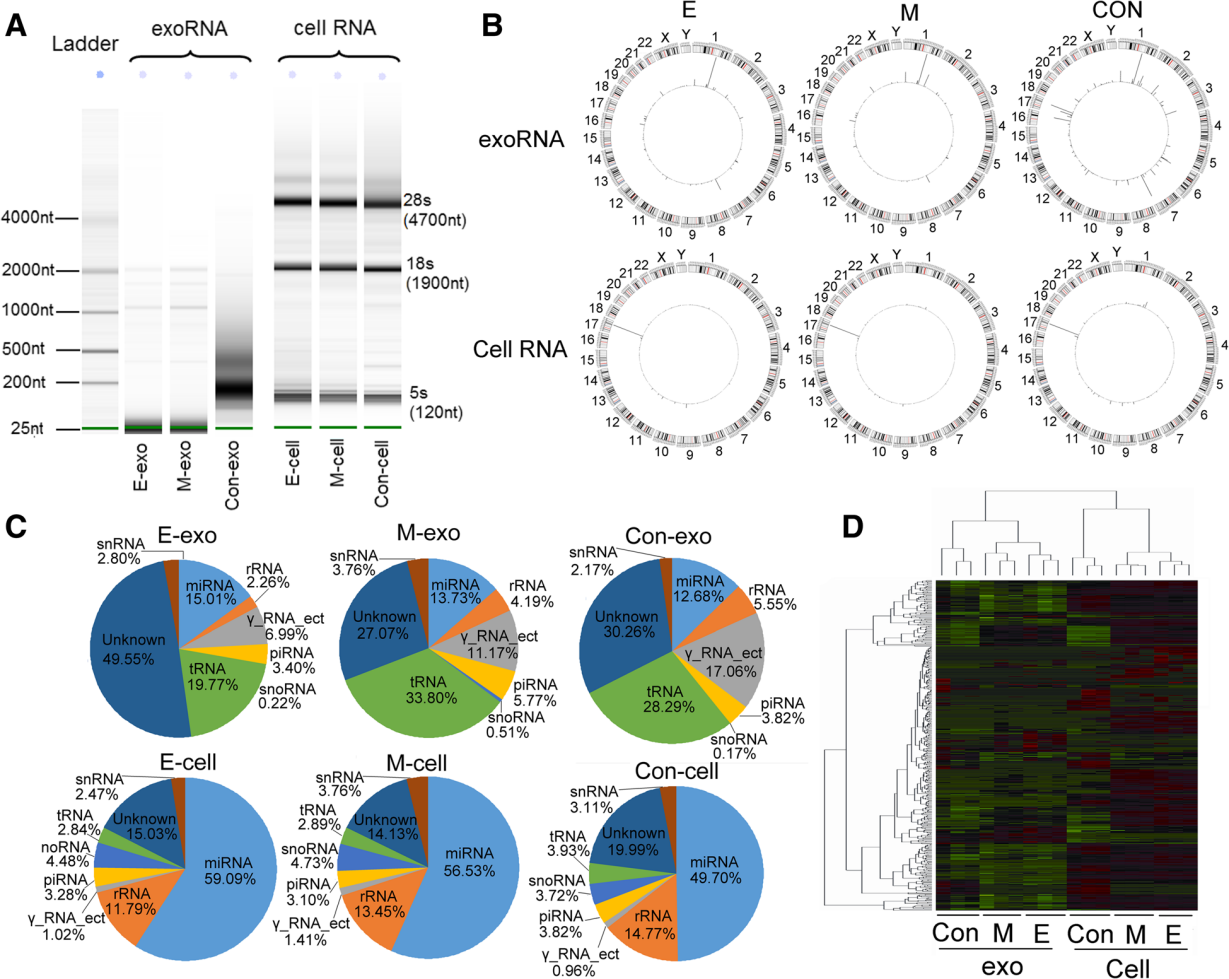
Comparison of small RNA profiles from cells and exosome extractions. **a** The length distribution of total RNA in cells and exosomes, as determined by Agilent RNA pico chip. (exo: exosomes for short) **b** All clean data was mapped by the National Center for Biotechnology Information (NCBI) to identify the chromosomal origin of small RNAs of interest. Outer rings display the ID of each chromosome, and the inner ring shows RNA abundance in the chromosomal region. **c** Pie chart summarizing the annotation of small RNA species. miRNA, rRNA and tRNA are the main identified RNAs in total distribution of small RNA. **d** Heatmap and cluster patterns of differentially expressed miRNAs among E/M/Con-exosomes and cells

To examine dynamic changes in the exosomal RNA (exoRNA) profile following EMT, high-throughput sequencing analysis was performed using RNA samples extracted from six groups: E-phenotype A549 cells (E-cells), exosomes derived from E-cells (E-exosomes), M-phenotype A549 cells (M-cells), exosomes derived from M-cells (M-exosomes), 16HBE cells (Con-cells) and exosomes derived from 16HBE cells (Con-exosomes) (Additional file 2: Figure S2A). A progressive decrease of E-marker and increase of M-marker levels from Con-cells, E-cells to M-cells indicated an appropriate and dynamic EMT model (Additional file 2: Figure S2B). The majority of exoRNA were small (< 200 nt), which differed from those of cells presenting specific ribosomal RNA (5S, 18S and 28S rRNA). In addition, there was variation in the RNA size-distribution between exosomes from A549 (E, M-exoRNA) and those from 16HBE (Con-exoRNA) (Fig. 2a).

Among the 18 samples, we obtained 228.28 million clean reads. An average of 78.38% of exosomal clean reads and 98.92% of cellular clean reads could be mapped to known RNAs of the human genome. Investigation of the chromosomal location of all the mapped sRNA revealed that the sRNA of exosomes mainly originated from chromosome 1, which was significantly different from the cell RNA location (mainly on chromosome 17). In addition to chromosome 1, many sRNA of Con-exoRNA were also found on chromosomes 7 and 17 (Fig. 2b).

The mappable sequences were annotated to miRNA and other small non-coding RNAs. The percentages of miRNA, rRNA, tRNA and other RNA were significantly different between exoRNA and cell RNA: miRNA and tRNA were the most abundant known sRNAs in exosomes, while miRNA and rRNA were the most abundant sRNA in cells. In addition, cells contained a higher percentage (49.7–59.09%) of miRNA than exosomes (10.40–13.47%) (Fig. 2c). To better demonstrate the variation among the groups, an unsupervised hierarchical clustering analysis was performed. As expected, the heat map showed a clear separation between exoRNA and cell RNA. Either in exoRNA or in cell RNA, more similar expression patterns were found between the E and M groups than between the E and Con groups or the M and Con groups (Fig. 2d).

### M-exosomes had distinct miRNA profiles which may be involved in EMT and cancer progression when compared with E-exosomes, con-exosomes or M-cells

**Fig. 3:**
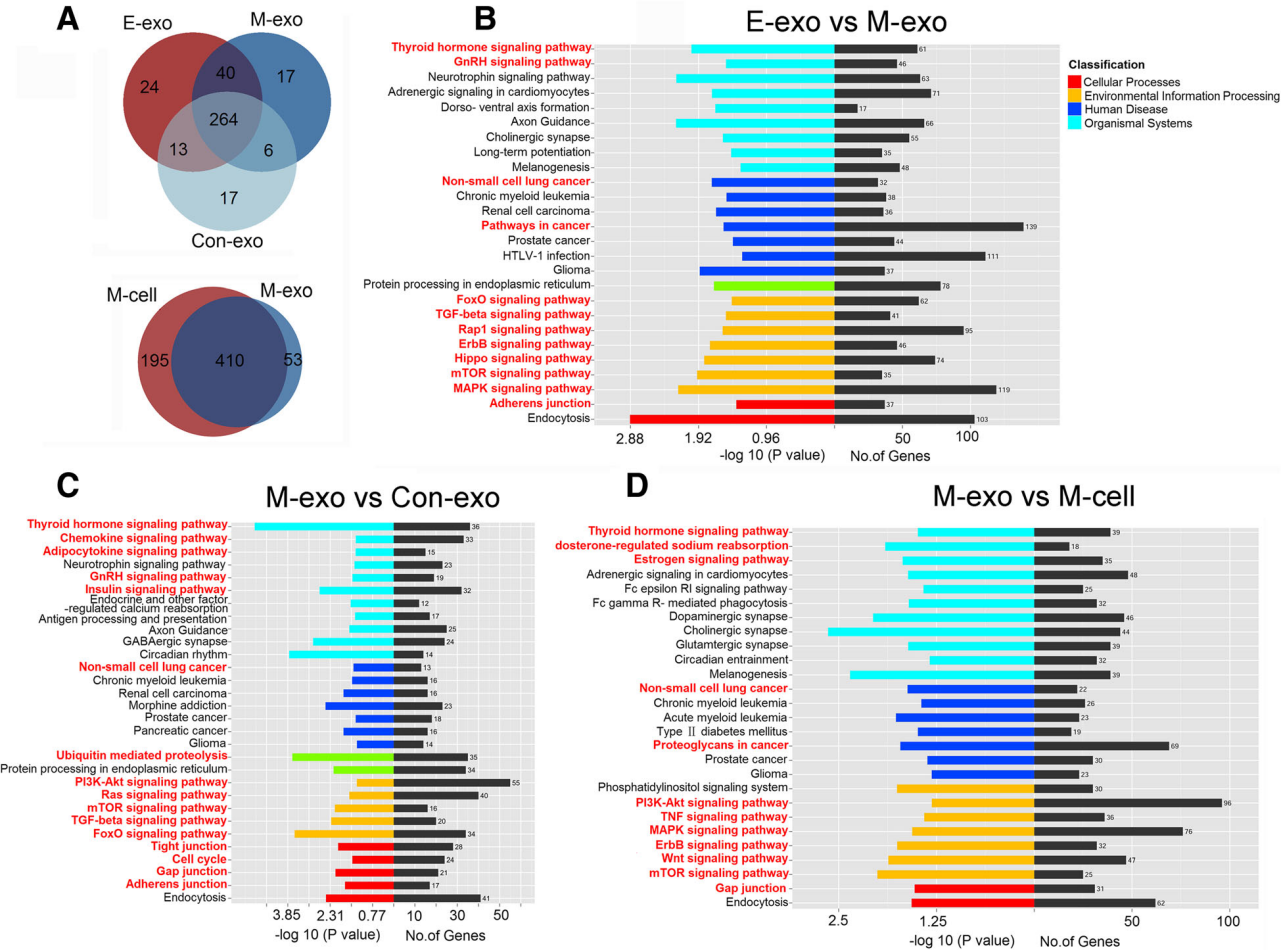
miRNA profiles of M-exosomes showed functional enrichment of tumor EMT and progression. **a** Venn diagram comparing the number of overlapping miRNAs among E/M/Con-exosomes, or between M-exosomes and M-cells. **b**, **c**, **d** The KEGG analysis of the predicted targets of different miRNAs between groups (M- vs E-exosomes, M- vs Con-exosomes, M-exosomes vs M-cells). Red labels highlight the targets which were enriched in pathway related to tumor progress

The contents of exosomes may change following EMT. First, we analyzed differences in the exosomal miRNA profiles among three archetypical phases of the EMT process (Con-exosomes, E-exosomes, M-exosomes). Of all detectable miRNAs, 264 miRNAs were common to E, M and Con-exosomes. A small amount of miRNAs were identified as unique for each group, such as hsa-miR-487b-3p, which was only detected in M-exosomes. (Fig. 3a). Regarding the differential level of miRNA expression among each group, 50, 137 and 199 miRNAs were screened out between M-exosomes and E-exosomes, M-exosomes and Con-exosomes, E-exosomes and Con-exosomes respectively. 10 of the most differentially expressed miRNAs were shown in Table 1. Additionally, the unique expressed miRNAs in M-exosomes compared with E-exosome, M-cell and Con-exosomes, were shown in Table 2.

**Table 1:** Top 10 significantly differentially expressed miRNAs between M-exo vs E-exo/Con-exo/M-cell

M-exo vs E-exo					
miRNA_ID	M-exo	E-exo	up/down	|log2(fold change)|	*P*-value
miR-26a-5p	342.32	161.34	up	1.09	2.10E-13
miR-92b-3p	65.09	219.15	down	1.75	1.64E-11
miR-193a-5p	16.66	69.67	down	2.06	3.14E-11
miR-424-3p	15.12	68.51	down	2.18	1.43E-10
miR-181b-5p	139.99	62.18	up	1.17	2.40E-09
miR-143-3p	101.23	41.20	up	1.30	3.62E-09
miR-125a-5p	110.20	282.86	down	1.36	2.32E-08
miR-193b-5p	27.52	103.43	down	1.91	2.63E-08
miR-92a-3p	1115.32	2646.67	down	1.25	1.10E-07
miR-3184-3p	641.22	2490.33	down	1.96	1.33E-07
M-cell vs E-cell					
miRNA_ID	M-exo	E-exo	up/down	|log2(fold change)|	*P*-value
miR-143-3p	476.7317	15.61803	up	4.931893	1.04E-244
miR-181a-2-3p	225.878	62.4662	up	1.854396	9.44E-59
miR-4483	21.115	117.744	down	2.479313	1.16E-21
miR-145-5p	15.63757	0	up	17.25466	1.05E-14
miR-4521	38.8255	123.3244	down	1.667382	6.41E-13
miR-1180-3p	119.0278	254.7887	down	1.098002	8.95E-11
miR-494-3p	47.59617	22.741	up	1.06555	2.24E-09
miR-654-3p	44.7572	20.77677	up	1.107149	2.64E-09
miR-365a-5p	48.06983	23.8603	up	1.01052	8.64E-09
miR-370-3p	27.4212	9.933367	up	1.464937	1.48E-08
M-exo vs Con-exo					
miRNA_ID	M-exo	Con-exo	up/down	|log2(fold change)|	*P*-value
miR-205-5p	8.22	4634.82	down	9.14	2.31E-188
miR-200c-3p	10.84	2762.23	down	7.99	1.45E-63
miR-192-5p	6328.30	700.17	up	3.18	4.67E-56
miR-148a-3p	243.13	6603.26	down	4.76	1.15E-55
miR-10a-5p	289.49	16.94	up	4.09	2.83E-52
miR-203a-3p	22.32	395.25	down	4.15	3.08E-40
miR-381-3p	83.07	3.12	up	4.74	8.54E-34
miR-584-5p	0.00	67.33	down	19.36	1.46E-33
miR-409-3p	44.56	1.92	up	4.54	2.96E-17
miR-127-3p	65.47	5.16	up	3.67	1.45E-18
E-exo vs Con-exo					
miRNA_ID	E-exo	Con-exo	up/down	|log2(fold change)|	*P*-value
miR-205-5p	7.21	4634.82	down	9.33	3.58E-137
miR-10a-5p	561.77	16.94	up	5.05	7.58E-75
miR-148a-3p	321.91	6603.26	down	4.36	5.40E-62
miR-192-5p	8828.24	700.17	up	3.66	1.64E-51
miR-200c-3p	11.53	2762.23	down	7.90	3.65E-49
miR-203a-3p	25.61	395.25	down	3.95	3.58E-38
miR-409-3p	76.15	1.92	up	5.31	1.04E-35
miR-26a-5p	161.34	1570.80	down	3.28	7.03E-35
miR-584-5p	0.43	67.33	down	7.30	4.54E-34
miR-193a-5p	69.67	1.66	up	5.39	7.01E-34
M-exo vs M-cell					
miRNA_ID	M-exo	M-cell	up/down	|log2(fold change)|	*P*-value
miR-1290	687.15	26.38	up	4.70	2.42E-49
miR-320c	83.16	19.92	up	2.06	2.65E-49
miR-1246	1104.80	185.33	up	2.58	7.24E-49
miR-4497	362.06	2.97	up	6.93	6.46E-47
miR-146b-5p	137.29	40.88	up	1.75	4.34E-43
miR-7704	42.93	2.45	up	4.13	2.40E-35
miR-5787	42.62	0.00	up	18.70	4.57E-21
miR-423-5p	163.74	61.46	up	1.41	1.31E-17
miR-7641	17.91	0.26	up	6.11	2.14E-16
miR-4532	11.44	0.00	up	16.80	7.88E-13

The expression levels were showed by the number of reads per million clean tags (RPM). The mean values of three triplicate experiments showed in the table. The edgeR bioconductor package was used to analyze the difference between groups and calculate the *P* values

**Table 2:** miRNAs only detected in M-exosomes in paired-comparisons

M-exo vs E-exo							
miRNA_ID	M-exo			E-exo			*P*-value
	R1	R2	R3	R1	R2	R3	
miR-487b-3p	1.34	2.47	2.70	0	0	0	0.017447
M-exo vs Con-exo							
miRNA_ID	M-exo			Con-exo			*P*-value
	R1	R2	R3	R1	R2	R3	
miR-452-5p	10.68	18.02	20.42	0	0	0	7.11E-13
miR-493-3p	7.05	20.25	18.95	0	0	0	6.27E-12
miR-935	6.92	17.61	18.87	0	0	0	1.67E-11
miR-300	7.12	16.90	18.30	0	0	0	2.79E-11
miR-889-3p	5.04	11.09	8.91	0	0	0	2.56E-07
miR-654-5p	2.75	10.27	8.58	0	0	0	2.82E-06
miR-31-3p	4.16	4.75	12.74	0	0	0	3.39E-06
miR-2682-5p	3.69	9.80	6.45	0	0	0	9.40E-06
miR-485-5p	2.15	5.11	4.33	0	0	0	0.001321
miR-370-3p	1.28	4.11	3.51	0	0	0	0.004795
M-exo vs M-cell							
miRNA_ID	M-exo			M-cell			*P*-value
	R1	R2	R3	R1	R2	R3	
miR-5787	60.90	30.93	36.03	0	0	0	4.57E-21
miR-4532	11.41	6.81	16.09	0	0	0	7.88E-13
miR-4488	6.58	10.39	8.82	0	0	0	2.55E-10
miR-4508	7.99	5.63	8.66	0	0	0	4.91E-09
miR-4492	7.59	4.99	9.31	0	0	0	8.76E-09
miR-1273 g-3p	5.51	6.63	8.01	0	0	0	3.66E-08
miR-4516	5.17	2.29	6.13	0	0	0	7.01E-06
miR-184	0.87	5.93	4.49	0	0	0	0.000158
miR-199a-5p	1.14	2.23	6.05	0	0	0	0.000596
miR-217	0.87	3.40	4.17	0	0	0	0.001142

Exosomes are capable of altering the recipient cell phenotype by carrying RNA cargoes, such as miRNAs. Whether miRNA contained in M-exosomes can influence tumor development, including the EMT process, needs to be studied. To investigate the function of these differentially expressed miRNAs, KEGG enrichment pathway analysis was performed, including target genes of all the miRNAs that were differentially expressed between M-exosomes and E-exosomes, and the top 20 differentially expressed miRNAs between M-exosomes and Con-exosomes. The significant pathways are shown in Fig. 3b and c. To our surprise, besides pathways involved in exosome formation, transport, and other physiologic functions, the most enriched pathways were related to tumor progression, such as classical signaling pathways (ErbB, mTOR, FoXO, MAPK, PI3K-Akt, etc.), metabolic processes involved in cancers (adipocytokine and insulin signaling pathways, ubiquitin mediated proteolysis, aldosterone regulated sodium reabsorption), hormones and proteins related to cancers (thyroid hormone, gonadotropin-releasing hormone (GnRH), chemokine signaling pathway, proteoglycans in cancer) and some highly attractive functions directly associated with EMT (TGF-β, tight junctions, gap junctions, adherence junctions, etc.).

MiRNA profiles of exosomes resemble those of their parent cells. However, the top 10 different miRNAs of E- and M-exosomes were almost different with the top 10 different miRNAs of E- and M-cells (Table 1). Some miRNAs like miR-5787, miR-4532 and miR-4488 were only selectively packaged into exosomes (Table 2) or up-regulated in M-exosomes when compared with M-cells (Table 1). Intriguingly, these miRNAs may target genes involved in EMT and cancer progression (Fig. 3d).

### Exosomes derived from M-phenotype cancer cells promote the transformation of recipient cells from E-phenotype to M-phenotype

**Fig. 4:**
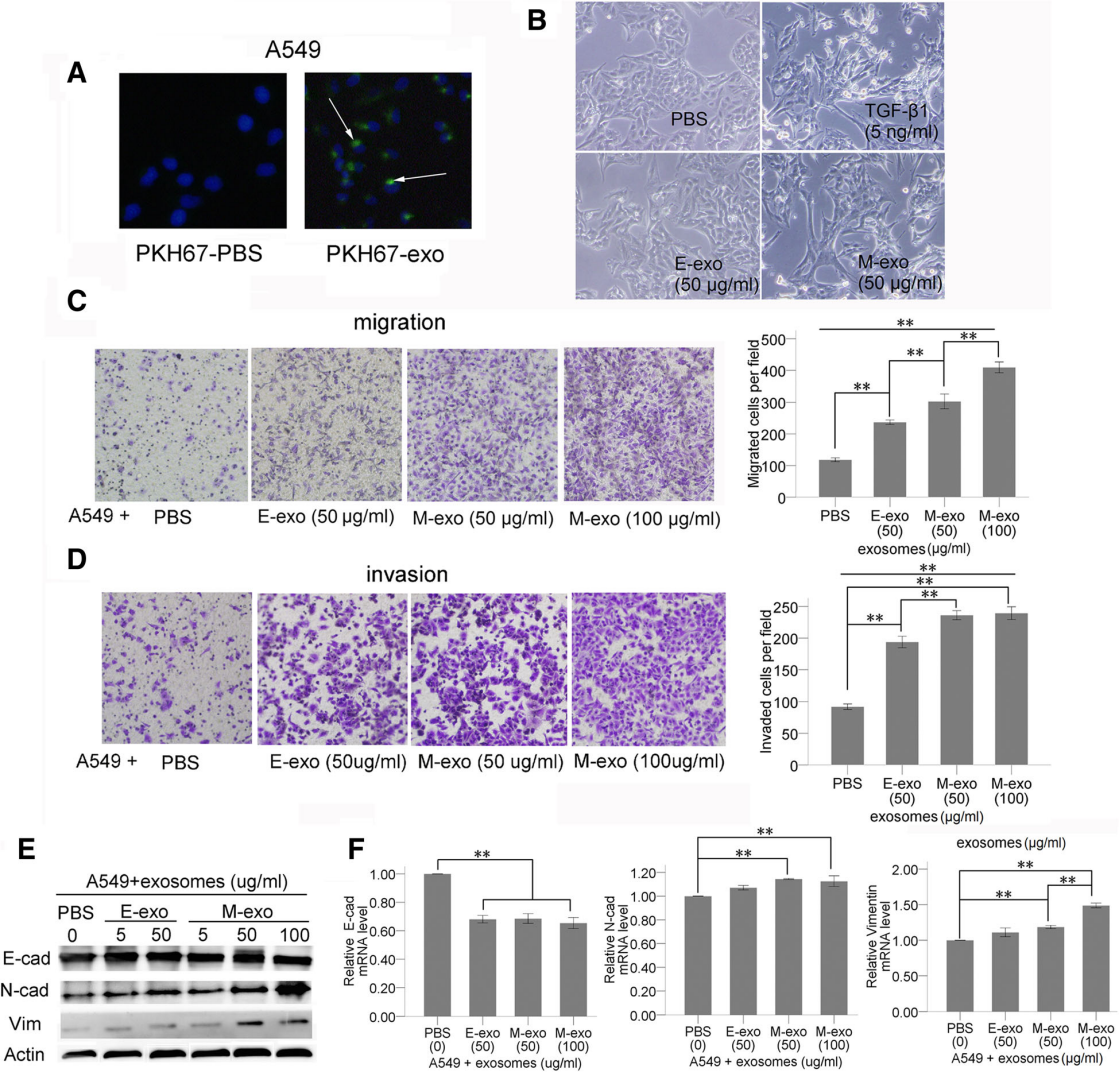
Functional studies of E/M-exosomes in A549 cells **a** Immunofluorescence microscopy revealed that green PKH67-labeled exosomes were readily taken up and incorporated into cultured A549, as shown by DAPI staining. Obvious green fluorescence signal appeared around the blue fluorescence stained nuclei of A549. **b** Morphological cellular changes of epithelial A549 cells upon M-exosome delivery. **c** The migrated cell numbers were significantly different between four groups with different treatments. **d** Matrigel invasion assays demonstrated the same trend. **e** E/M-exosomes induce an increase of N-cadherin and vimentin protein levels in A549 cells. Upregulated expression became more pronounced after co-culture with M-exosomes than with E-exosomes. However, no effects of exosomes could be found on E-cadherin expression. **f** mRNA level of N-cadherin and vimentin was only significantly upregulated after M-exosome stimulation, indicating M-exosomes showed stronger influence than E-exosomes. (*P < 0.05, **P < 0.01)

The above results showed that differentially expressed miRNAs contained in exosomes may target genes related to EMT and the progression of cancer. Therefore, the function of exosomes coming from different cell types was verified by a co-culture experiment. First, lipid staining and immunofluorescence microscopy were used to confirm that exosomes derived from A549 cells can enter the same recipient tumor cells (A549) (Fig. 4a). Then we found the morphology of epithelial A549 cells turned from being round into a spindle-like mesenchymal phenotype and lost intercellular junctions after treatment with M-exosomes (Fig. 4b).We next examined the effect of E- and M-exosomes on migration and invasion of E-phenotype A549 cells using a transwell system. When compared with the PBS –treated group, although both E- and M-exosomes enhanced the migration and invasion of A549 cells, the effect of M-exosomes was more noticeable than that of E-exosomes (Fig. 4c, d). Additionally, the increased capacity of migration and invasion, EMT is characterized by a variation in EMT-related markers. Regarding the epithelial marker E-cadherin, although no significant change was found at the protein level, the mRNA level decreased in A549 cells when they were co-cultured with both E- and M-exosomes. However, protein and mRNA expression of N-cadherin and vimentin were significantly up-regulated in A549 cells when co-cultured not with E-exosomes but with M-exosomes (Fig. 4e, f). Similarly, up-regulated expression of vimentin became more pronounced after treatment with a higher concentration of M-exosomes (Fig. 4f).

**Fig. 5:**
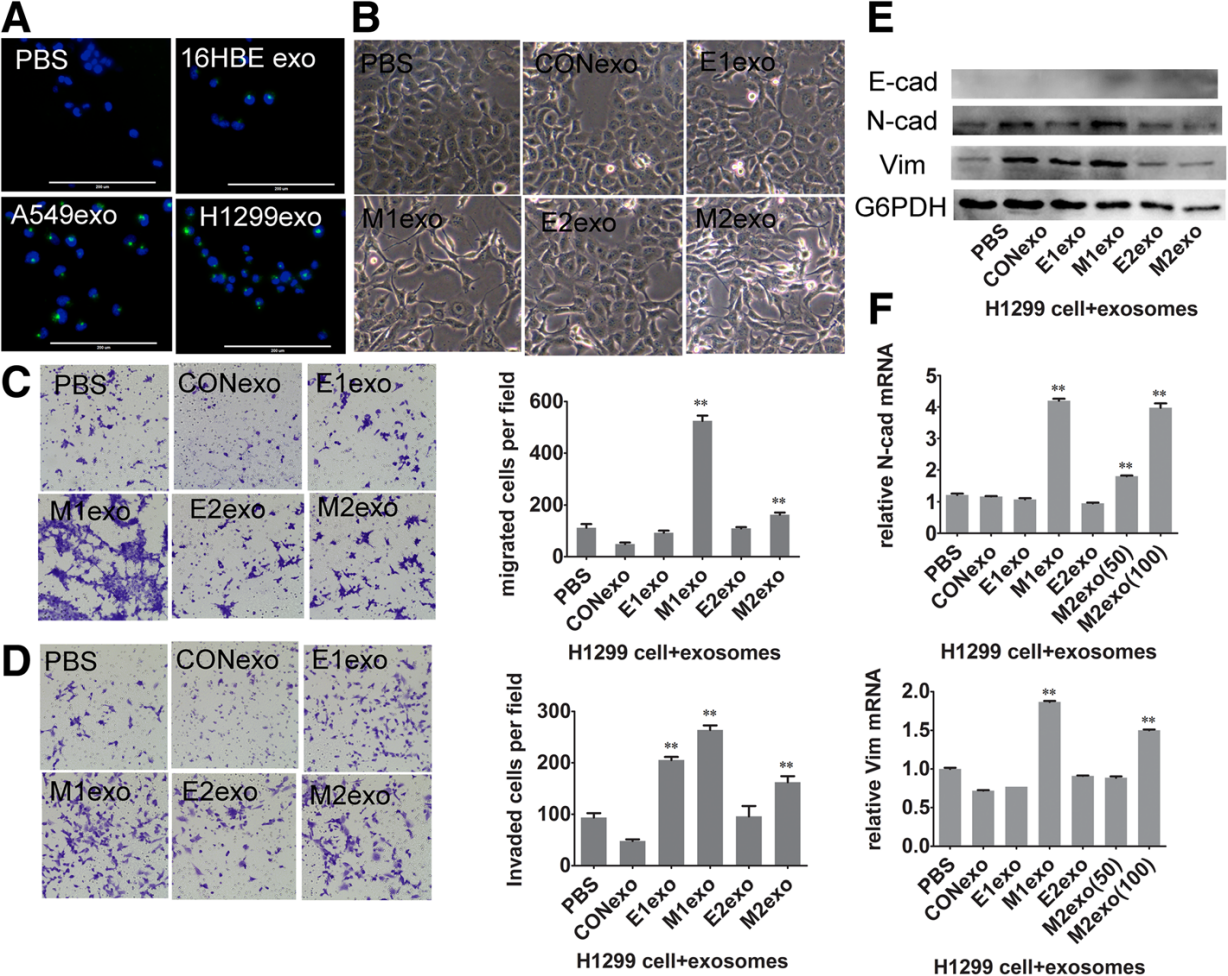
Effect of treatment with E/M-exo in lung cancer cell lines H1299. **a** Exosomes derived from 16HBE, A549 and H1299 were taken up by H1299 cells. **b** Morphology of H1299 cells changed from E- to M- phenotype after M1-, E2- and M2-exo treatment. **c** The migrated cells numbers were significantly higher in the group with M1- and M2-exo treatment. **d** More invaded cells were found in E1, M1 and M2-exo treatment groups. **e** M1-exo induced an increase in N-cadherin and vimentin in H1299 cells. **f** The mRNA level of N-cad and vimentin and was significantly upregulated after M1- and M2-exosome (100μg/ml) stimulation. (CON-exo, E1-exo, M1-exo, E2-exo, M2-exo represent exosomes derived from 16HBE, E-phenotype A549 cells, M-phenotype A549 cells, E-phenotype H1299 cells, M-phenotype H1299 cells, respectively). (*P < 0.05, **P < 0.01)

To determine the effect of E/M-exosomes in other lung cancer cell lines, H1299 with higher malignant behavior than A549 was selected for functional analysis. In the intake test, exosomes from 16HBE, A549, H1299 can be taken by H1299 cells (Fig. 5a). Morphologically, H1299 cells turned from being round into a spindle-like mesenchymal phenotype after stimulation by M1/E2/M2-exosomes (Fig. 5b). In the transwell system, more migrated H1299 cells were found in M1 and M2-exosome treated groups (Fig. 5c), and more invaded cells were observed in E1, M1 and M2-exosomes (Fig. 5d) than PBS and Con-exosomes treated groups. M-exosomes showed higher ability to induce invasion and migration than E-exosomes for both A549 and H1299. In addition to the expression of EMT markers, the protein (Fig. 5e) and mRNA levels (Fig. 5f) of N-cadherin and vimentin of M1-exosomes group were higher than PBS, Con and E1-exosomes groups. The mRNA levels of N-cadherin (both 50 and 100 μg/ml concentration) and vimentin (only 100μg/ml) of the M2-exosome group were higher than those for the PBS, Con and E2-exosomes groups (Fig. 5f).

**Fig. 6:**
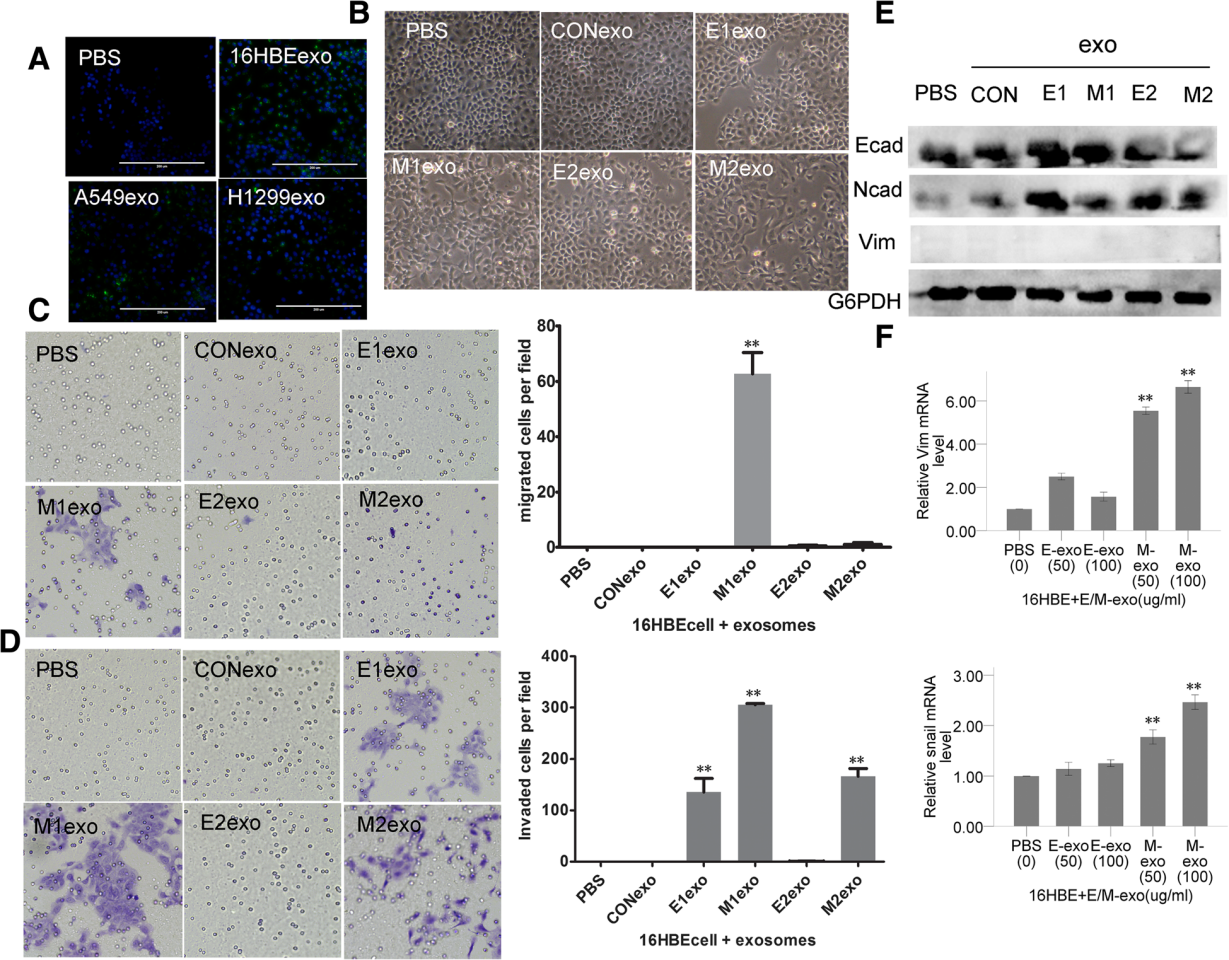
Effect of treatment with E/M-exosomes in 16HBE recipient cells. **a** Exosomes derived from 16HBE, A549 and H1299 were taken up by 16HBE cells. **b** Morphological cellular changes of epithelial 16HBE cells upon E/M-exosomes delivery. **c** The migrated cell numbers were significantly higher in the group with M1-exo treatment. **d** In matrigel invasion assays, more invaded cells were found in E1, M1 and M2-exo treatment groups. (Considering that 16HBE were non-tumor cells and were harder to pass through the chamber coated with matrigel, we extended the incubation time to 60 h). **e** E/M-exosomes induced an increase in N-cadherin in 16HBE cells. Protein levels of E-cadherin became more pronounced after co-culture with E2- and M2-exosomes than other groups. **f** E/M-exosomes from A549 cells affected the mRNA expression of EMT-related markers in 16HBE cells. The mRNA level of vimentin and snail were significantly upregulated after M-exosome stimulation, and M-exosomes showed stronger influence than E-exosomes. (*P < 0.05, **P < 0.01)

Given that exosomes derived from A549 and H1299 cells could also be taken up by 16HBE cells (Fig. 6a), EMT-related function and markers of 16HBE cells were measured after co-culturing with E/M-exosomes to investigate whether the M-exosomes could induce EMT of non-tumorigenic respiratory epithelial cells. Consistent with the results for cancer cells, M-exosomes showed greater potential to enhance the mesenchymal morphological changes (Fig. 6b) and the invasion and migration abilities of 16HBE cells (Fig. 6c, d) than E- and Con-exosomes. The 16HBE cells treated with E1/M1/E2/M2-exosomes also showed increased protein expression of the mesenchymal marker, N-cadherin, when compared with PBS/Con-exosomes treated cells (Fig. 6e). Down-regulated expression of E-cadherin became more pronounced after co-culture with E2 and M2-exosomes than other groups (Fig. 6e). Interestingly, the mRNA levels of vimentin and snail were more markedly elevated in 16HBE cells after E/M-exosomes treatment compared with that in A549 as acceptor cells (Fig. 6f).

## Discussion

Exosomes serve as molecular messengers by delivering various effectors or signaling macromolecules between specific cells; it is possible that changes in their production may contribute to the development and progression of cancer. A key initiating step in cancer metastasis involves EMT [27, 28], and recent evidence highlights the emerging role of exosomes in EMT of cancer [12]. Proteomic analysis has shown that changes in cellular differentiation status (E/M phenotype) translate into unique qualitative rearrangements in the protein cargo of exosomes [18, 19]. However, no study has reported changes in the overall exosomal miRNA expression profile in the EMT process. Our study revealed changes in the exosomal miRNA profile upon EMT. Using bioinformatics and an exosome–cell co-culture experiment, we demonstrated that the altered miRNAs identified in the exosomes may facilitate EMT, migration, and invasion of acceptor cells.

The small RNA profiles of E/M/Con-exosomes were compared to screen out differentially expressed miRNAs between exosomes from mesenchymal (M) cells and those from epithelial (E/Con) cells (Table 1). For example, one of the best studied pathways of miRNA-mediated EMT inhibition is that of the miR-200 family and miR-205-related [29–31]. In our study, miR-200c and miR-205 levels in exosomes from invasive cells (M-cells) was significantly decreased in comparison with those from non-invasive cells (Con-cells), indicating the ability of M-exosomes to maintain malignant characteristics of the parental cells. MiR-10a plays a role in the EMT process in glioma [32]. A higher level of miR-10a was found in M-exosomes than in Con-exosomes, which may be related to lung cancer cell oncogenesis and the EMT process. However, the expression characteristics of some miRNAs in the exosomes in our study disagree with those in previous studies. Our results revealed the selective enrichment of miR-26a in exosomes derived from highly invasive M-cells in comparison with that in E-cells. Given that miRNA-26a has been demonstrated to act as a tumor- [33] and EMT-suppressor [34, 35], this may be explained by an exosome-mediated clearance mechanism by which exosomes may permit highly invasive tumor cells to release tumor suppressors from the cells, thereby maintaining their tumorigenic phenotype [12, 36]. However, whether exosomal miRNAs play different biphasic roles in different cancers or stages, and the exact mechanism requires further investigation.

To our surprise, with regard to the function of these screened-out miRNAs, although many of them have been rarely reported in EMT-related research, KEGG pathway analysis identified that these components have the potential to drive signal transduction networks in EMT and cancer progression. Firstly, in our research, the miRNA-targeted genes were found to play key roles in hormone-related pathways such as GnRH [37, 38], insulin [39], estrogen and the thyroid hormone signaling pathway. Previous studies have shown that GnRH and insulin have a correlation with lung cancer. Over-expression of estrogen-related receptor alpha (ERRα) promoted the EMT of A549 cells, and down-regulated the epithelial markers and up-regulated the mesenchymal markers [40]. Thyroid hormone can efficiently up-regulate TGF-β mRNA expression in breast cancer [41] and hepatocellular carcinoma [42]. Based on these reports and the KEGG pathway analysis in our research, we presume that the miRNA contained in M-exosomes may promote EMT and invasion of recipient cells via these cancer-related hormones. Secondly, target genes of the differentially expressed miRNAs were enriched in metabolism-related pathways such as the insulin signaling pathway, adipocytokine signaling pathway, aldosterone-regulated regulated sodium reabsorption and ubiquitin-mediated proteolysis. Our findings suggest that tumors may reprogram pathways of nutrient metabolism through exosomes to meet the bioenergetic and redox demands of malignant cells [43, 44]. Intriguingly, glypican-1 (GPC1), a cell-surface proteoglycan, was specifically enriched on cancer-cell-derived exosomes and this was correlated with tumor burden and the survival of patients with pancreatic cancer [45]. Our results indicated that selective enrichment of miRNAs by M-exosomes can target the proteoglycan pathway in cancer. It is possible that the miRNAs contained in exosomes may be an indirect mechanism by which exosomes regulate cancer-related proteoglycans. Most importantly, M-exosomes carrying the miRNA complex could control the microenvironment through the activation of TGF-β, ErbB, Wnt, mTOR, PI3K-Akt, FoXO, Ras, and MAPK signaling pathways and cell junction (adherens, tight and gap junction)-remodeling pathways to promote EMT and metastasis of acceptor cells. In conclusion, our observations support the hypothesis that secreted miRNAs enclosed within exosomes derived from cells of an aggressive phenotype may play a pivotal role in tumor progression and EMT by regulating gene expression and affecting the function of targeted cells.

Although most components of the exosomes were similar to those of the originating cells, several studies indicate that part of the RNA “cargo” of exosomes is significantly different from that of the parental cell [46, 47]. As a consequence of sorting, the functional properties and biological role of exosomes may differ from those of their parental cells. In our study, RNA size distribution, chromosomal location, and small RNA constituent ratio of exosomes differed from those of the originating cells. More importantly, the specific up-regulated miRNAs in M-exosomes can target genes enriched in pathways involved with gap junctions, non-small-cell lung cancer, and tumor progression. The specific contents of exosomes may be transferred from tumor cells to stromal cells and endothelial cells, and thus affect tumor invasion, angiogenesis, metastasis, and drug resistance [48, 49]. We propose that exosomes produced by M-cells may obtain an aggressive phenotype from their parental cells, and that miRNAs specifically expressed in M-exosomes associated with EMT and metastasis may promote transfer of the malignant phenotype to epithelial recipient cells.

To facilitate tumor growth and metastasis, exosomes derived from tumor cells can modify their microenvironment [50, 51] or directly transfer molecules related to the malignant phenotype to other cancer cells [52], such as Epidermal Growth Factor Receptor (EGFR) [53] and drug resistance genes [54–57]. Several studies have demonstrated that exosomes derived from invasive cancer cells can induce EMT in recipient cells [15]. Using miRNA profile analysis, we found that the differentially expressed miRNAs of M-exosomes were related to pathways of EMT and tumor progression. Based on our sequencing study, it was thought to be worth investigating whether M-exosomes containing special small RNA cargos can contribute to the induction of EMT in recipient cells. Intriguingly, our data showed that M-exosomes can be taken up by cancer or non-tumorigenic epithelial cells, and cause alterations in marker expression and cell behavior (migration and invasion) associated with EMT. Exosomes can transfer invasion and metastasis factors [58] to uninfected cells or communicate with surrounding stromal cells [58] to produce cellular changes characteristic of tumor progression. Given that M-phenotype cells are associated with increased invasive potential, we found that this malignant phenotype could be transferred by exosomes in our study. Tumor cells can transfer exosomes to distant sites via the circulatory system, leading to cell transformation and tumor development [4]. Our research presents a new insight into the role of M-exosomes, which may transform lung cancer cells with a more invasive phenotype and cause distant metastasis.

Of note, apart from the difference between M-exosomes and Con-exosomes, we also found different miRNA profiles of E-exosomes and Con-exosomes. The top 10 differentially expressed miRNAs between E- and Con-exosomes were almost the same as those for M- vs Con-exosomes, though the ranking of miRNA expression level was different (Table 1). The specific miRNA profile of E-exosomes may also drive the same signal transduction networks in EMT and cancer progression as M-exosomes. So, we speculated that not only the more malignant M-exosomes, but also E-exosomes from general tumor cells have carcinogenic capacity. That may explain the relatively smaller change in EMTness observed following treatment with exogenous E-exosomes compared to that following treatment with M-exosomes.

miRNA in exosomes plays an important role in the development and progression of cancer [25]. Previous studies have reported single or several special miRNAs that may relate to EMT [23]. It is well known that cells can secrete signaling molecules to recipient cells. However, when a more complex “message” needs to be sent, cells use exosomes [5]. Therefore, the change of exosome “cargo” following EMT may not just refer to one miRNA. In this study, for the first time, we considered the miRNA cargo as a whole to investigate the profile changes following EMT by high-throughput sequencing. We established that changes in cellular E/M status translate into unique qualitative rearrangements in the miRNA cargo of exosomes. Again, transferred miRNAs may be associated with EMT, migration, and invasion when they target gene expression in recipient cells. In addition, considering the complexity of exosome contents, we should clarify that not only the alterations in miRNA profiles, but also of other molecules like protein and DNA may contribute to the ability of M-exosomes to induce EMT in target cells; this is worth further investigation.

Exosomes are secreted in CCM, and are also found naturally in blood and other bodily fluids [4]. Evidence suggests that exosomes derived from serum obtained from patients with late-stage lung cancer induce EMT in non-cancerous recipient cells, when compared with healthy serum derived exosomes [17]. Several authors have noted that the miRNA content of circulating exosomes can be used as diagnostic markers [17, 59]. Therefore, in further clinical translation research, the miRNA in circulating exosomes following EMT, identified in our study, may serve as a source of new biomarkers to detect EMT-like processes in lung cancer.

## Conclusions

This study indicated that changes in the exosomal miRNA profile upon EMT, and the miRNAs specifically expressed in exosomes produced by mesenchymal cells are associated with EMT and metastasis, and may promote transfer of the malignant (mesenchymal) phenotype to epithelial recipient cells. These miRNAs may serve as a source of new biomarkers to detect EMT-like processes in lung cancer.

## Additional files

Additional file 1: **Figure S1.** Particles isolated from CCM by ExoQuick were identified by TEM, western blot and NTA. (A) Particles with lipid bilayer structure and right size around 100 nm were observed by TEM. Scale bars for 100 nm. (B) Exosome markers (CD9, CD63, TSG101) expressed in exosomes derived from E/M-A549 cells, while non-exosomal markers (calnexin) didn’t exist in exosomes. (C) NTA profile of E and M exosomes: The y-axis was the number of particles/ml (in millions per milliliter) and the x-axis was the diameter of the particles (unit: nm). (D) No significant differences in total number and overall size distribution of the exosomes were found between E and M groups. (TIF 1983 kb)

Additional file 2: **Figure S2.** (A) A flowchart of sequencing group preparation. E: A549 cells treated with PBS, M: A549 induced with 5 ng/ml TGF-β1 for 48 h, and 16HBE: human bronchial epithelial cells. The experiment on each group was repeated three times and 18 RNA samples were obtained. The sequencing triplicates done at the experimental level (triplicate experiments) rather than the sequencing level (three runs with the same library). (B) The E/M phenotype of the sequencing cells was verified by the expression level of EMT markers. (TIF 361 kb)

## Abbreviations

Con-cells: 16HBE cells
Con-exosomes: Exosomes derived from 16HBE cells
E-cells: Epithelial cells
E-exosomes: Exosomes derived from epithelial cancer cells
EMT: Epithelial-mesenchymal transitions
exoRNA: RNA in exosomes
M-cells: Mesenchymal cells
M-exosomes: Exosomes derived from mesenchymal cancer cells
sRNA: Small RNA
TGF-β1: Transforming growth factor-β1
