# Correction: Alterations in exosomal miRNA profile upon epithelial-mesenchymal transition in human lung cancer cell lines

**DOI:** 10.1186/s12864-023-09810-7

**Published:** 2023-12-07

**Authors:** Yue-Ting Tang, Yi-Yao Huang, Jing-Huan Li, Si-Hua Qin, Yong Xu, Tai-Xue An, Chun-Chen Liu, Qian Wang, Lei Zheng

**Affiliations:** 1 Department of Laboratory Medicine, Nanfang Hospital, Southern Medical University, No.1838 North Guangzhou Avenue, Guangzhou, 510515 Guangdong China; 2 Department of Clinical Laboratory, Zhujiang Hospital, Southern Medical University, Guangzhou, Guangdong China; 3 Department of Clinical Laboratory, Zhongnan Hospital, Wuhan University, Wuhan, Hubei China; 4 Department of Physiology, Anatomy and Genetics, University of Oxford, Oxford, Oxfordshire UK

**Correction: BMC Genomics 19, 802 (2018)**

**https://doi.org/10.1186/s12864-018-5143-6**

Following publication of the original article [1], an error was reported in Fig. 1C. The GAPDH band from the upper panel was inadvertently reused in the lower panel. Following a thorough reevaluation of the raw data, the authors have identified the correct GAPDH band, which has now been substituted in the lower panel in the corrected Fig. 1.

**Fig. 1:**
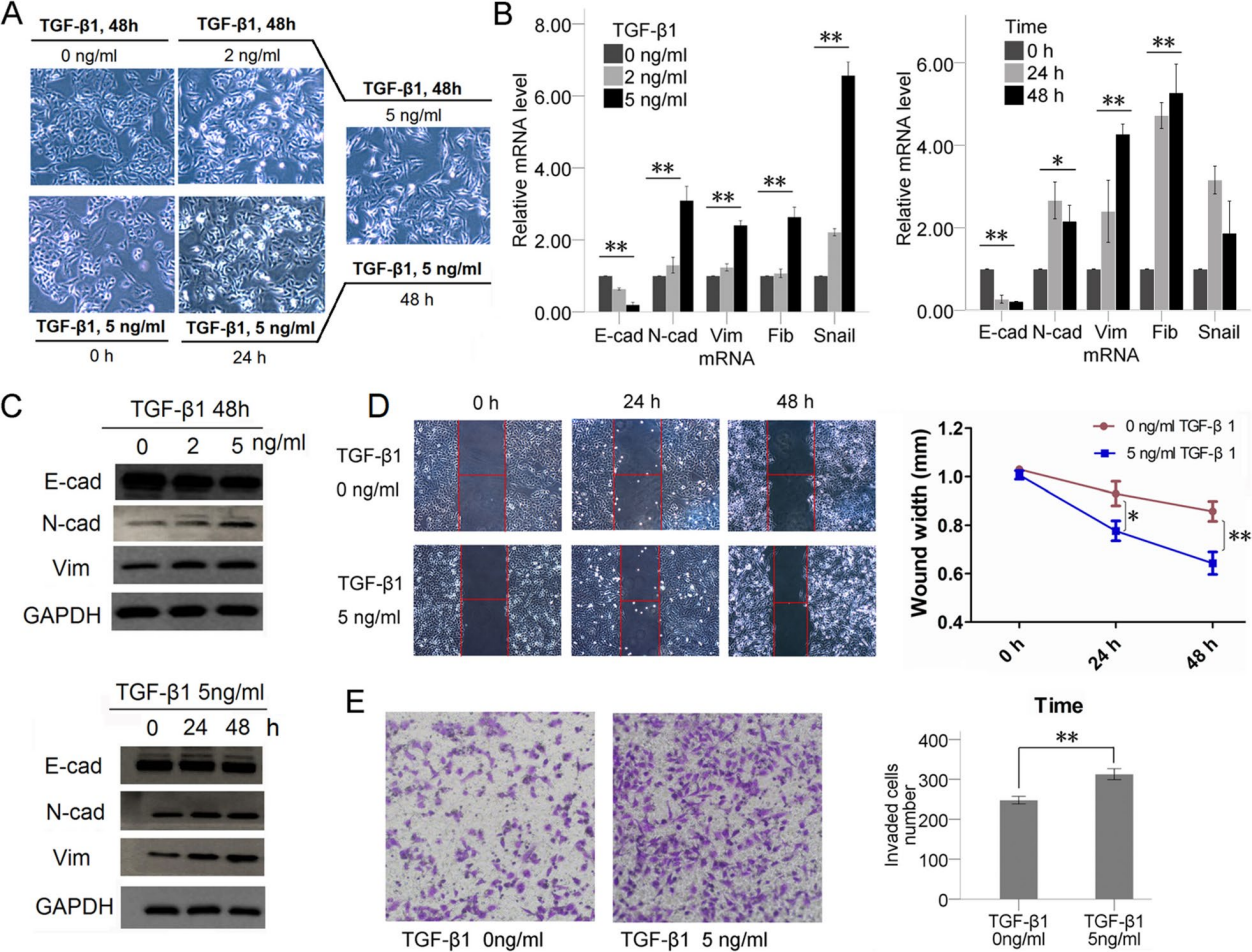
TGF-β1 was used to establish EMT cell models. **a** Morphology of A549 cells changed from E- to M- phenotype after TGF-β1 treatment. The mRNA (**b**) and protein (**c**) levels of EMT-related markers of A549 cells changed after being induced by TGF-β1. TGF-β1 significantly reduced E-phenotype marker (E-cadherin (E-cad)) levels, but increased the M-phenotype marker (N-cadherin (N-cad), vimentin (Vim), fibronectin (Fib) and snail) levels in a TGF-β-concentration-dependent manner but not a strict time-dependent manner. **d** The wound healing assays proved that TGF-β1 treatment can significantly increase cell migration abilities. The wound widths were significantly shorter at 24 h after TGF-β1 treatment than in the no-treatment group (*P* < 0.05), and this difference was more significant after 48 h treatment (*P* < 0.01). **e** Invasion assays were used to determine cell invasion. Invaded cell numbers were significantly higher in TGF-β1 treatment than no-treatment groups, indicating TGF-β1 can improve cell invasion ability

In Fig. 1A, the authors employed a common group to assess the impact of TGF-β1 stimulation at a concentration of 5ng/ml over a 48-hour duration (depicted as the same image in both upper and lower panels in the original article). To ensure clarity and avoid any potential misinterpretation of the data, the authors display only one picture for this group in the updated Fig. 1A, simplifying the representation from the two found in the original version. This modification is intended to improve the figure's overall comprehensibility, based on suggestions from fellow researchers.

The corrected Fig. 1 is presented here for reference.
