# Supplementary figures and images for: Alterations in exosomal miRNA profile upon epithelial-mesenchymal transition in human lung cancer cell lines
Source: BMC Genomics. 2018 Nov 6;19:802. doi: 10.1186/s12864-018-5143-6 (PMC6219194; doi:10.1186/s12864-018-5143-6)

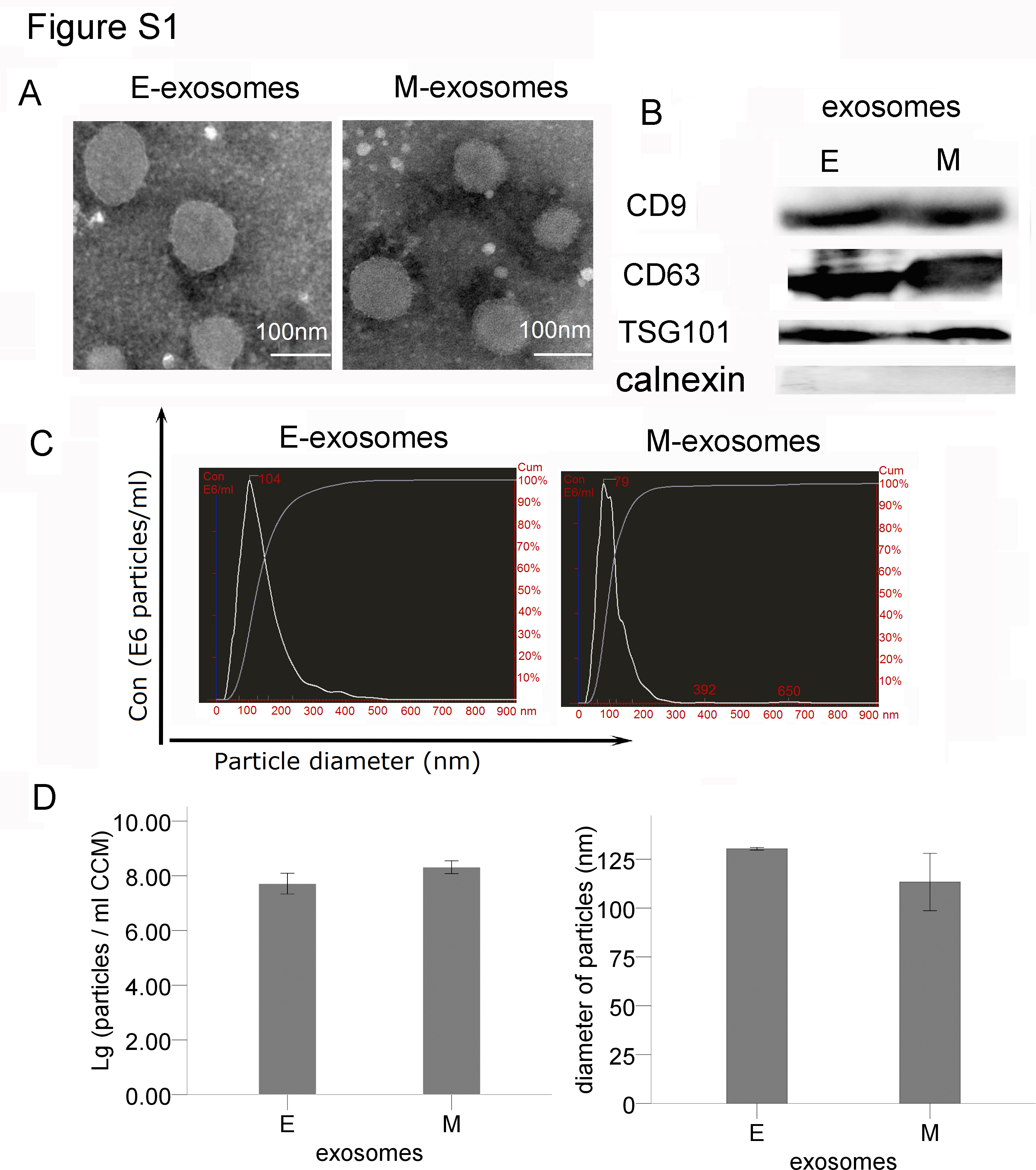

Supplement: Supplementary file 1 — Figure S1. Particles isolated from CCM by ExoQuick were identified by TEM, western blot and NTA. (A) Particles with lipid bilayer structure and right size around 100 nm were observed by TEM. Scale bars for 100 nm. (B) Exosome markers (CD9, CD63, TSG101) expressed in exosomes derived from E/M-A549 cells, while non-exosomal markers (calnexin) didn’t exist in exosomes. (C) NTA profile of E and M exosomes: The y-axis was the number of particles/ml (in millions per milliliter) and the x-axis was the diameter of the particles (unit: nm). (D) No significant differences in total number and overall size distribution of the exosomes were found between E and M groups. (TIF 1983 kb) [file 12864_2018_5143_MOESM1_ESM.tif]

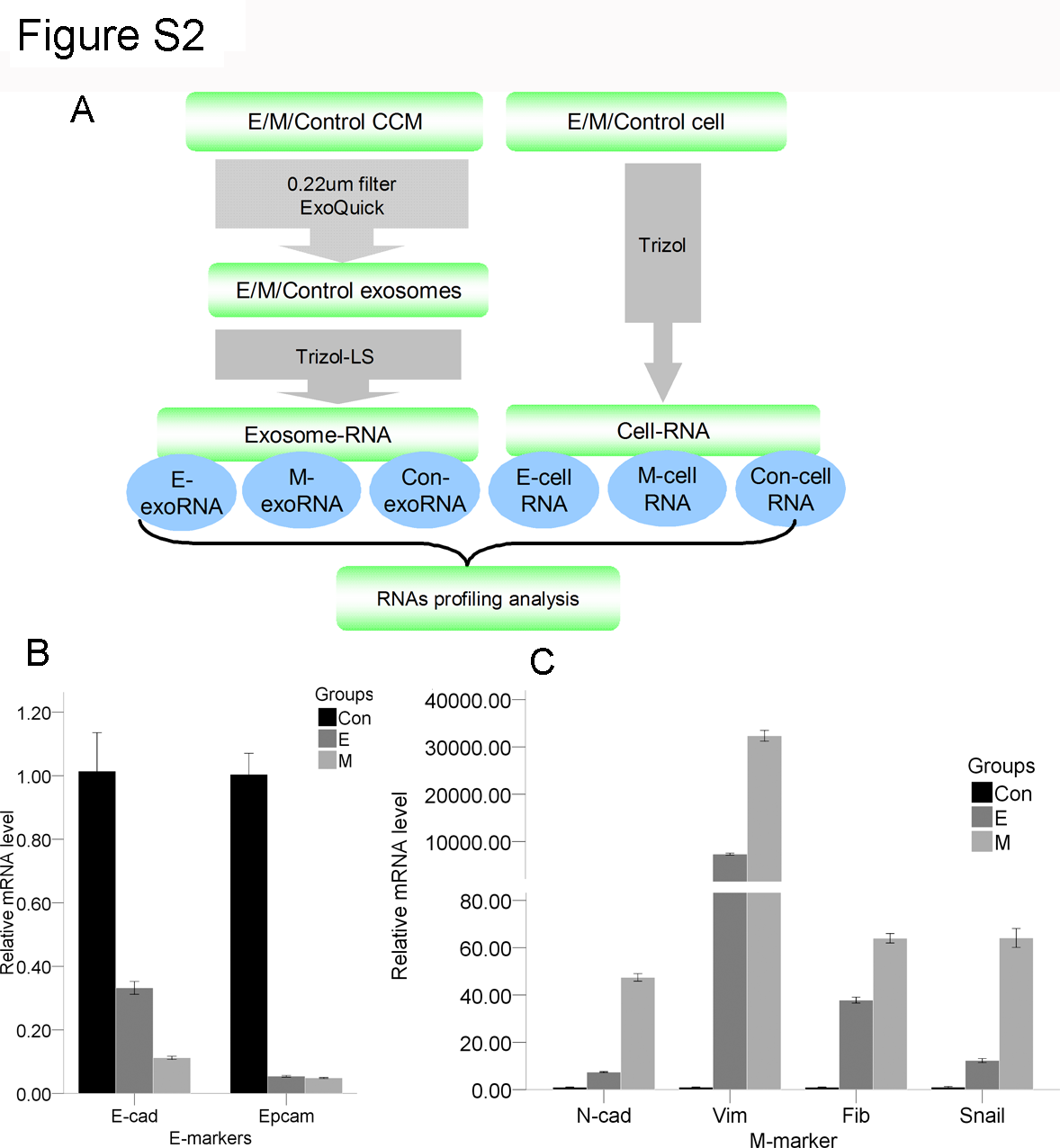

Supplement: Supplementary file 2 — Figure S2. (A) A flowchart of sequencing group preparation. E: A549 cells treated with PBS, M: A549 induced with 5 ng/ml TGF-β1 for 48 h, and 16HBE: human bronchial epithelial cells. The experiment on each group was repeated three times and 18 RNA samples were obtained. The sequencing triplicates done at the experimental level (triplicate experiments) rather than the sequencing level (three runs with the same library). (B) The E/M phenotype of the sequencing cells was verified by the expression level of EMT markers. (TIF 361 kb) [file 12864_2018_5143_MOESM2_ESM.tif]
